# In Silico Screening of Therapeutic Targets as a Tool to Optimize the Development of Drugs and Nutraceuticals in the Treatment of Diabetes *mellitus*: A Systematic Review

**DOI:** 10.3390/ijms25179213

**Published:** 2024-08-25

**Authors:** Ana Francisca T. Gomes, Wendjilla F. de Medeiros, Isaiane Medeiros, Grasiela Piuvezam, Juliana Kelly da Silva-Maia, Ingrid Wilza L. Bezerra, Ana Heloneida de A. Morais

**Affiliations:** 1 Nutrition Postgraduate Program, Center for Health Sciences, Federal University of Rio Grande do Norte, Natal 59078-970, RN, Brazil; aft.gomes00@gmail.com (A.F.T.G.); wendjilla_1@hotmail.com (W.F.d.M.); juliana.maia@ufrn.br (J.K.d.S.-M.); 2 Biochemistry and Molecular Biology Postgraduate Program, Biosciences Center, Federal University of Rio Grande do Norte, Natal 59078-970, RN, Brazil; isaianemedeirosss@gmail.com; 3 Public Health Postgraduate Program, Center for Health Sciences, Federal University of Rio Grande do Norte, Natal 59078-970, RN, Brazil; gpiuvezam@yahoo.com.br; 4 Department of Nutrition, Center for Health Sciences, Federal University of Rio Grande do Norte, Natal 59078-970, RN, Brazil; ingrid.bezerra@ufrn.br

**Keywords:** computer simulation, molecular dynamics simulation, molecular docking simulation, therapeutics, designer drugs, drug development, Diabetes mellitus

## Abstract

The Target-Based Virtual Screening approach is widely employed in drug development, with docking or molecular dynamics techniques commonly utilized for this purpose. This systematic review (SR) aimed to identify in silico therapeutic targets for treating Diabetes mellitus (DM) and answer the question: What therapeutic targets have been used in in silico analyses for the treatment of DM? The SR was developed following the guidelines of the Preferred Reporting Items Checklist for Systematic Review and Meta-Analysis, in accordance with the protocol registered in PROSPERO (CRD42022353808). Studies that met the PECo strategy (Problem, Exposure, Context) were included using the following databases: Medline (PubMed), Web of Science, Scopus, Embase, ScienceDirect, and Virtual Health Library. A total of 20 articles were included, which not only identified therapeutic targets in silico but also conducted in vivo analyses to validate the obtained results. The therapeutic targets most frequently indicated in in silico studies were GLUT4, DPP-IV, and PPARγ. In conclusion, a diversity of targets for the treatment of DM was verified through both in silico and in vivo reassessment. This contributes to the discovery of potential new allies for the treatment of DM.

## 1. Introduction

The computer-based approach, often referred to as the ‘in silico’ approach, enables the investigation of disease-related targets through bioinformatics. It involves the construction of molecules for potential therapeutic use, optimization of molecular compatibility, and the simulation of interactions [1,2,3]. Some advantages of this approach include environmental sustainability, as it reduces the use of solvents, minimizes electronic waste disposal, and lowers gas emissions. Additionally, it aligns with the fundamental principles of the 3Rs (replacement, reduction, and refinement) by mitigating the use of animals in experiments. Furthermore, it saves time, reduces financial costs, and allows the analysis of chemical and physical characteristics [4,5,6].

This highlights the growing prominence of this type of research as a trend in the development of therapeutic candidates, such as nutraceuticals terms derived from “nutrition” and “pharmaceutical”. Nutraceuticals are bioactive compounds obtained from natural sources, such as food, that can be therapeutic agents for the treatment of diseases, in contrast to drugs or pharmaceuticals that are substances derived from synthetic sources, demonstrating the applicability of technological advancements across various approaches. This includes strategies for planning preclinical studies, which bridge the gap between theory and experimental research [4,7,8,9].

Several techniques are employed in in silico studies, with docking and molecular dynamics being common in the Target-Based Virtual Screening (TBVS) approach, also known as structure-based virtual screening. This approach leverages structural information about the target protein or receptor and facilitates the discovery of new drugs and alternative therapeutics for the treatment of diseases [4,10].

Molecular docking is one of the most used structure-based in silico methods that predicts the binding modes and affinity of small molecules and the target receptor. The process includes sampling conformations of all possible binding modes, generating several poses that represent the ligand’s position within the active site of the protein, and ranking these conformations by assigning scores to distinguish the correct poses from the incorrect ones. This allows the estimation of the interaction affinity between the protein and the ligand, helping to identify the most likely binding mode [11,12,13]. Despite providing valuable insights for predicting molecular interactions, this technique is often complemented by other bioinformatics approaches and experimental analyses [14,15].

Molecular dynamics simulates the functional interaction between molecules and ligands at the atomic level over time, predicting the behavior of each atom in the protein, as well as the processes of conformational change, ligand binding and protein folding [16,17]. It takes into account the force on each atom to determine the position and speed of the atoms later; therefore, it is a dynamic simulation [16]. There are also techniques that can complement simulations such as dimerization free energies that describe the strength of interaction of different molecules, thermodynamic integration (TI) or End-point free energy calculation methods such as Molecular Mechanics/Poisson Boltzmann Surface Area (MM/PBSA) or Molecular-Mechanics/Generalized Born Surface Area (MM/GBSA), free energy perturbation (FEP) that are good accurate [18,19].

TBVS is one kind of computer-aided drug design (CADD) that allows the identification of targets involved in diseases, verification of the binding site, and assessment of toxicity [20,21]. The utilization of CADD is steadily increasing and has contributed to the discovery of new molecules, drugs, and nutraceuticals for the control of diseases. CADD encompasses various aspects, including sequencing, structural prediction, and understanding functionality, as well as the mode of interaction with therapeutic targets [1,4]. Molecular docking and molecular dynamics are widely used techniques, particularly in a structure-based approach, which is one of the methods used in CADD [13,16].

In this context, these analyses can be valuable for future studies aimed at prospecting therapeutic targets for various diseases [22]. One of these diseases is Diabetes mellitus (DM), characterized by alterations in insulin secretion, action, or both [23]. According to estimates from the IDF Diabetes Atlas, the prevalence of people diagnosed with diabetes was 536.6 million in 2021, with projections indicating an increase to 783.2 million by 2045. This suggests a growing diabetes epidemic that will continue to expand, affecting an increasing portion of the population worldwide [24].

Through in silico analyses, several studies aim to identify the interaction between targets and therapeutic agents for treating DM [25,26,27,28]. Additionally, given the increasing use of in silico studies within the scientific community, systematic reviews (SR) are being conducted to compile the data from studies that employ this approach [29,30,31]. Prominent examples of in silico applications in medical research encompass a wide range of scenarios. These include the discovery of molecules for tuberculosis treatment, followed by subsequent in vitro or in vivo assessments to determine their potential impact. Additionally, innovative approaches such as drug repurposing through molecular docking to combat COVID-19 have emerged. Another compelling avenue is the utilization of in silico methodologies as efficient screening tools in the search for new drug candidates derived from flavonoid sources [29,30,31].

Currently, antidiabetic medications include sulfonylureas and glinides, which stimulate insulin secretion by the pancreas; alpha-glucosidase inhibitors, which slow the absorption of carbohydrates; biguanides, which reduce glucose release by the liver; glitazones, which enhance the incretin effect driven by the hormones GLP-1 (glucagon-like peptide 1), and GIP (gastric inhibitory peptide), both of which are glucose-dependent. Additionally, sodium/glucose cotransporter type 2 inhibitors prevent glucose reabsorption in the kidneys [32].

Identifying the therapeutic targets relevant to the treatment of DM through in silico studies that subsequently present validation through analyses in an in vivo study underscores the effectiveness of these targets’ utilization and lends support to their selection for future research endeavors aimed at treating diseases. Thus, gathering the data in a systematic review (SR) can help the scientific community and boost the discovery of new promising therapeutic agents. The objective of this SR was to identify the therapeutic targets investigated in in silico studies for the treatment of DM.

## 2. Methods

### 2.1. Protocol and Registration

The protocol for preparing the SR followed the recommendations established by the Preferred Reporting Items Checklist for Systematic Review and Meta-Analysis Protocols (PRISMA-P) [33]. The protocol was registered on the Prospective International Systematic Reviews platform (PROSPERO) under the number CRD42022353808, available at URL https://www.crd.york.ac.uk/prospero/display_record.php?ID=CRD42022353808 (accessed on 26 August 2022), and published in the PloS One journal under the title “In silico structure-based designers of therapeutic targets for Diabetes mellitus or obesity: A protocol for systematic review” [34]. It is worth mentioning that the registered and published protocol is related to the elaboration of two SRs, one addressing DM and the other addressing obesity, and the results of SR for DM will be presented in this article.

### 2.2. Research Question

The research question was established according to the PECo strategy (P—problem; E—exposure; Co—context) (Table S1), being defined as “What therapeutic targets have been used in in silico analyses for the treatment of Diabetes mellitus?” as registered in PROSPERO (CRD42022353808) and published in the SR protocol article [34].

### 2.3. Eligibility Criteria

#### 2.3.1. Inclusion Criteria

Studies that analyzed antidiabetic therapeutic targets were included (P—problem), used in the treatment of DM (E—exposure), being original studies in silico that evaluate the molecular dynamics and/or molecular docking (Co—context).

#### 2.3.2. Exclusion Criteria

Studies with therapeutic targets that participate in the control of other diseases were excluded (P—problem), focusing on other comorbidities or complications arising from DM (E—exposure) and that were exclusively in vivo, in vitro, or in silico, other types of in silico studies that do not correspond to molecular dynamics and/or molecular docking (Co—context). Studies that used therapeutic targets from a non-human organism were excluded. As a way of ensuring the effectiveness of using these targets in a biological environment, having greater scientific support, and increasing the degree of evidence, studies that did not reassess therapeutic targets by performing in vivo analyses were also excluded. Furthermore, letters to the reader, preprint works (preprint), review articles, dissertations, theses, conference abstracts, gray literature, and book chapters were also excluded.

### 2.4. Search Strategy

The articles for the SR were searched through electronic search in the following databases: Medline (PubMed), Web of Science, Scopus, Embase, ScienceDirect, and Virtual Health Library. A comprehensive search was performed without time or language restrictions, using combinations of MESH and EMTREE terms in the databases and a combination of Boolean operators (AND and OR). A manual search was performed when necessary to insert articles that might not have been included according to the search strategies by reading the references of the studies included in the SR. The search equations considering the PECo strategy are presented in Table S2. The strategies for searching for studies retrieved all studies published until 18 October 2022.

### 2.5. Study Selection

All articles were imported into the Rayyan application (version 0.1.0) for the screening step [35]. This platform facilitated the removal of duplicate studies.

The papers were read independently by two reviewers. Potentially eligible works were selected by reading the title and abstract for the next step, which consisted of the complete reading of these articles. In case of disagreements, a third evaluator joined for the resolution.

Reasons for exclusion from studies were recorded. The results of the selection or exclusion of studies were demonstrated using the flowchart recommended by the Preferred Reporting Items for Systematic Reviews and Meta-Analyses (PRISMA) [36].

### 2.6. Data Extraction

Data extraction was performed independently, also by two reviewers, and the following characteristics of the articles were extracted: authors, year, model (in silico), technique used (docking and molecular dynamics), docking score or potential interaction energy, amino acids with greater interaction and main outcomes as therapeutic targets, therapeutic agent used, in vivo evaluation of the therapeutic target, potential applications, and other timely data. For papers that were not found in full, contact was made via email (at least two attempts) to the respective authors. Data from the included articles were entered into a Microsoft Excel ® version 2016 (Natal, Brazil) software spreadsheet in a table designed to better select the necessary information.

### 2.7. Data Analysis and Synthesis

The results were presented in a summary table and in narrative form to describe the characteristics of the included studies. The included studies were structured around the therapeutic targets used in the treatment of DM, studied through in silico simulations with molecular dynamics or molecular docking, as well as three-dimensional models of these molecules or compounds used in interactions by computer simulations. To help organize the references, the Mendeley ® version 2.120.1 (Natal, Brazil) software was used [37]. No meta-analysis was carried out due to the methodology of the evaluated studies.

### 2.8. Risk of Bias

Two reviewers independently assessed the risk of bias and study quality. Discrepancies were resolved by a third evaluator. As there is no standardized tool for assessing the quality of in silico studies, a checklist developed and applied by Taldaev et al., was used [31] based on standardized guidelines for simulation Strengthening the Reporting of Empirical Simulation Studies (STRESS) [38]. Prior training was carried out to ensure uniformity in tool application.

## 3. Results

### 3.1. Selection and Characteristics of the Studies

**Figure 1:**
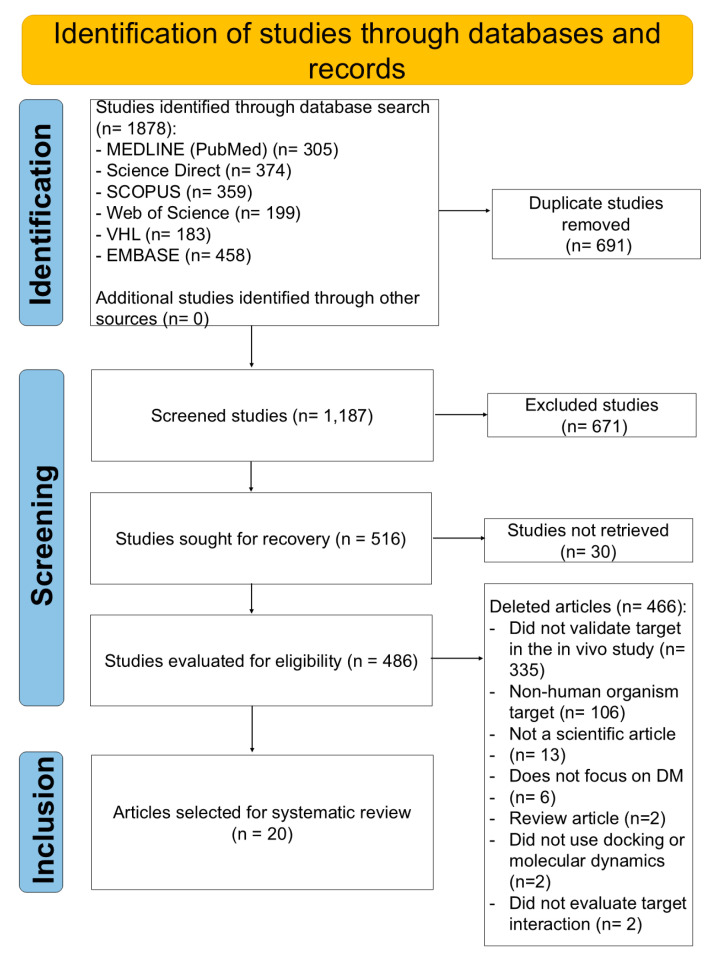
Flowchart of selection of articles included for the Systematic Review based on PRISMA [36].

Based on the search strategies employed to address the initial question, “What therapeutic targets have been used in in silico analyses for the treatment of diabetes mellitus?”, 1878 articles were initially retrieved. After removing duplicate articles, the titles and abstracts of 1187 studies were read, with 671 articles being excluded at this stage because they presented exclusion criteria such as not addressing therapeutic targets in the studies and being related to other diseases and other types of studies, other than in silico, in addition to some specificities of scientific publications already mentioned. For the complete reading stage, 516 articles remained; however, 30 of these articles were not found in full (the corresponding authors were contacted via email at least twice, but no responses were obtained). Therefore, 486 full articles were read, of which 466 articles were excluded according to the criteria presented in Figure 1. After all these steps, 20 articles were included in the SR (Figure 1).

This analysis included studies published in English between 2012 and 2023. These studies revealed a total of eleven in silico therapeutic targets, which were: glucose transporter 4 (GLUT-4), dipeptidyl peptidase IV (DPP-IV), peroxisome proliferator-activated receptor gamma (PPARγ), protein kinase B/AKT (AKT), glucagon-like peptide-1 (GLP-1), α-amylase, glucose-dependent insulinotropic polypeptide (GIP), insulin receptor substrate 1 (IRS1), glycogen synthase kinase 3 (GSK-3), insulin receptor (IR), phosphatidylinositol 3-kinase signaling pathway (PI3K), and insulin-like growth factor (IGF-1).

Most of the 3D structures for the therapeutic targets were determined experimentally using the X-ray diffraction method from the Protein Data Bank (PDB) files. These structures had resolutions ranging from 1.62 Å to 2.90 Å. A few studies utilized therapeutic target structures obtained through homology modeling, accessible via resources provided by UniProt Consortium (Table S3).

The most frequently studied therapeutic target was GLUT-4, investigated in seven of the included studies. Following closely were DPP-IV, PPARγ, and AKT, each examined in six studies. GLP-1 was the subject of two studies, while α-amylase, GIP, IRS1, GSK-3, IR, PI3K, and IGF-1 were the focus of one study each.

Among the articles included, 16 studies exclusively used molecular docking as a technique for the in silico study, while four works used both docking and molecular dynamics. The key findings from the articles referring to the in silico studies were presented in summary form (Table S4) [39,40,41,42,43,44,45,46,47,48,49,50,51,52,53,54,55,56,57,58]. The studies did not perform statistics on the results obtained from the in silico analyses.

In terms of in vivo validations of the therapeutic targets analyzed in silico, male *Wistar* rats (n = 11) were the most used, followed by male *Sprague Dawley* mice (n = 2) and male *C57BL/6J* mice (n = 2). The remaining studies utilized Wistar albino rats (n = 1), albino rats of both sexes (n = 1), male *Charles Foster* rats (n = 1), *Wistar* rats without specifying sex (n = 1), and male *Goto–Kakizaki* (GK) rats (n = 1).

Of the in vivo analyses of therapeutic targets, the most frequently used method, corresponding to the therapeutic target used in the in silico approach, was the analysis of mRNA expression (12 times). This was followed by an assay on serum samples (4 times), nuclear protein analysis (3 times), target plasma and serum concentration analysis (two times), and an assay on plasma samples. Colonic target concentration analysis was the least frequently used method, employed once in the studies.

When validating the therapeutic targets used in in silico studies through in vivo studies, it became evident that these investigated therapeutic targets are promising for the treatment of DM. The results showed improvements concerning the analyzed targets, elucidating the mechanism of action involved. Furthermore, they showed enhancements in commonly monitored DM parameters, such as fasting glucose, oral glucose tolerance, insulin, glycated hemoglobin, and HOMA-IR (Table S5) [39,40,41,42,43,44,45,46,47,48,49,50,51,52,53,54,55,56,57,58].

### 3.2. Bias Risk Assessment

**Figure 2:**
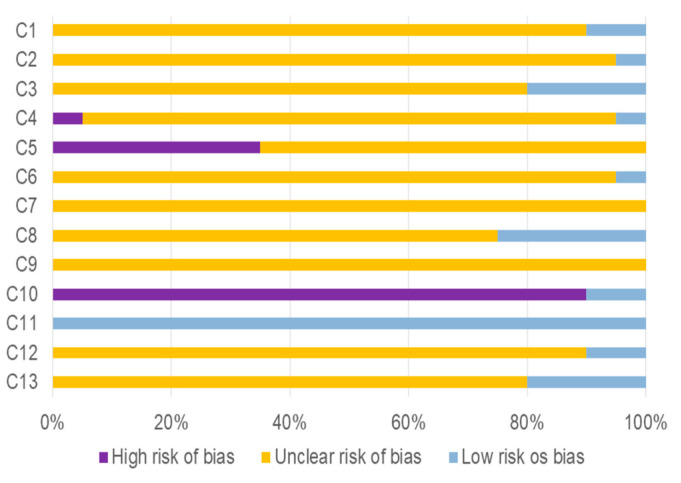
Risk of bias assessment of studies included in the systematic review, based on the checklist developed and applied by Taldaev et al. [31]. C (1–13): criterion.

The risk of bias assessment of the studies included in the SR was performed using the checklist developed and applied by Taldaev et al. [31]. It was observed that most studies did not provide complete information on methodological details regarding in silico analyses (Figure 2).

Most studies did not provide information on whether they performed ligand filtering (90%) (criterion 1), assessed ligand ionization (95%) (criterion 2), or generated energetically possible conformations of the ligands (80%) (criterion 3). Regarding the selection of the therapeutic target, the majority of studies did not mention the resolution of the structure of the target used (90%) (criterion 4) or an alternative method used for obtaining the structure of these target structures (65%) (criterion 5).

In terms of target optimization, 95% of the studies did not include information about the control of histidine protonation (criterion 6). None of these studies (100%) reported protonation details for amino acids following X-ray crystallography or cryogenic electron microscopy (criterion 7). Additionally, 75% of the studies did not mention whether missing residues and side chains were added after X-ray crystallography or cryogenic electron microscopy (criterion 8), and any study (100%) specified whether metals were added (criterion 9).

Regarding the software employed in molecular docking analyses, most studies (90%) did not use software considered the “gold standard” (glide or GOLD) (criterion 10). Concerning the evaluation of the results, all studies (100%) carried out the visual control of the complexes (criterion 11). This criterion stands out as the only one consistently addressed among the included studies. In terms of re-docking (criterion 12), 90% of studies did not report whether this procedure was performed. Finally, 80% of the studies did not provide information about verifying docking results through in vitro studies (criterion 13).

## 4. Discussion

Advancements in the technologies employed in bioinformatics studies have greatly improved our understanding of the mechanisms underlying the interaction between targets and their therapeutic agents [1]. This progress has the potential to accelerate the development of drug candidates, as it enables the rapid generation of substantial data, reduces costs, and provides clearer guidance for subsequent in vitro and in vivo studies [4]. Consequently, there is a high possibility of discoveries for the treatment of diseases such as DM.

**Figure 3:**
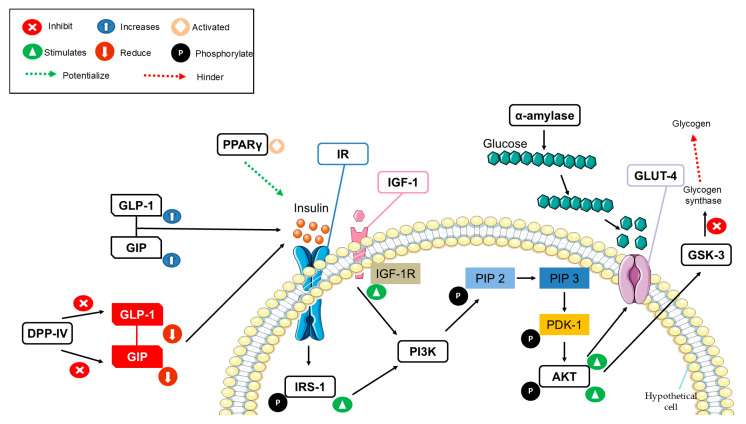
Diagram of a hypothetical cell with the main signaling pathways of therapeutic targets used in in silico studies for the treatment of diabetes mellitus. AKT: Protein kinase B/AKT; DPP-IV: Dipeptidyl peptidase IV; GIP: Gastric inhibitory peptide; GLP-1: glucagon-like peptide 1; GLUT-4: Glucose transporter 4; GSK-3: Glycogen synthase kinase 3; IGF-1: Insulin-like growth factor; IGF-1R: Insulin-like growth factor receptor; IR: Insulin receptor; IRS-1: Insulin receptor substrate; PI3K: Phosphatidylinositol 3-kinase; PIP2: Phosphatidylinositol 4,5-bisphosphate; PIP3: Phosphatidylinositol 4,5-triphosphate; PDK-1: 3-phosphoinositide dependent protein kinase 1; PPARy: Peroxisome proliferator-activated receptor gamma.

All the studies included in this SR examined therapeutic targets involved in multiple signaling pathways related to glycemic control (Figure 3). The regulation of these pathways can have a positive impact on the treatment of DM. The potential utility of these targets was identified through in silico studies and further corroborated through in vivo validation. This not only demonstrates their effectiveness but also increases the likelihood of their successful application in the treatment of DM.

One of the most frequently investigated targets in the studies covered by this SR was DPP-IV [39,42,50,52,53,55]. The DPP-IV is an oligopeptidase enzyme that belongs to the homodimeric serine peptidase family. It has the capability to cleave the N-terminal dipeptide residues of GLP-1 and GIP, resulting in the production of inactive incretins. This, in turn, leads to an insulinotropic function, ultimately causing higher postprandial glucose levels [59,60]. Inhibiting DPP-IV is considered one of the strategies for treating DM as it does not interfere with the actions of GLP-1 and GIP, thereby helping to maintain reduced glucose levels. Additionally, the SR also highlighted other therapeutic targets investigated in in silico studies. These targets are integral components of one of the most crucial pathways to glucose control, the insulin signaling pathway [61]. Notable among them are as follows: IR [48], IRS-1 [49], PI3K [54], Akt [41,44,49,54,57,58], and GLUT-4 [41,43,44,48,51,52,56].

Insulin has action mediated by the IR that is formed by two α subunits and two β subunits. Changes in its conformation occur upon binding to the extracellular region of the IR, triggering phosphorylation that activates tyrosine kinase and recruits IRS [62]. The IRs, responsible for organizing and mediating signaling complexes, subsequently undergo phosphorylation, leading to the activation of PI3K [63,64,65]. Activation of the protein AKT initiates the signaling cascade that results in the translocation of GLUT-4 to the cell surface, facilitating the transport of glucose into the cell [65,66,67].

PPAR-γ [41,43,44,45,50,52] also emerged as one of the most frequently studied targets in the SR. PPARs are receptor proteins functioning as transcription factors, with isoforms such as PPAR-α, PPAR-γ, and PPAR-β/δ. They regulate key metabolic pathways and are activated when a specific isoform of PPAR binds to its cognate ligand within the ligand-binding domain (LBD) [68,69]. Specifically, PPAR-γ is involved in glucose homeostasis and is considered an important target for treating DM [70,71]. Activation of PPAR-γ enhances insulin action in insulin-sensitive tissues, leading to reduced hepatic glucose production and increased peripheral glucose elimination [71]. Therefore, therapeutic agents acting as PPAR-γ agonists can be used as antidiabetic agents, as low levels of PPAR-γ have been associated with glucose metabolism abnormalities in islets [70,71,72].

Less studied according to SR, but also important, one of the therapeutic targets used in the in silico studies was α-amylase [46]. Amylases are enzymes responsible for cleaving the glycosidic bonds of carbohydrates, causing an increase in postprandial blood glucose [73,74]. Salivary α-amylase hydrolyses the α-1-4-glycosidic bond of starch, generating oligosaccharides or polysaccharides [75]. This enzyme is inactivated upon reaching the stomach, but another form is secreted by the pancreas and released in the small intestine to continue the hydrolysis process of saccharides, the pancreatic amylase [75]. While this enzyme becomes inactive upon entering the stomach, another form is secreted by the pancreas and released in the small intestine to continue the saccharide hydrolysis process—pancreatic amylase [75].

Other targets examined in in silico studies, as outlined in this SR, include GIP [47] and the GLP-1 [40,47]. These hormones are known as incretins and are released by the gastrointestinal tract, specifically by K cells (which secrete GIP) located in the duodenum and L cells (which secrete GLP-1) situated further down the gastrointestinal tract [76,77]. There is a stimulation of the autonomic nervous system immediately after ingestion of meals, which stimulates the release of these hormones at intestinal levels. They can increase insulin levels to control the increment in the postprandial glucose [76,77].

The GSK-3 was also investigated as a target in the studies included in the SR [52]. GSK-3 is a serine/threonine kinase that inhibits the action of glycogen synthase, hindering glycogen synthesis [78]. GSK-3 is overexpressed in the skeletal muscles of people with DM, reducing the ability of insulin to act in glucose control. Therefore, its inhibition can be beneficial for the treatment of DM [79].

Finally, IGF-1 was also a target examined in one of the included studies [54]. This hormone primarily operates within the endocrine system and is mainly secreted by the liver in response to Growth Hormone (GH). Its role extends to mediating the effects of GH on distant tissues. Additionally, IGF-1 plays a role in cellular growth, regulation, and viability through autocrine and paracrine functions. Dysregulation of IGF-1 can potentially have an impact on glucose homeostasis, affecting both insulin sensitivity and the function of pancreatic beta cells [80]. The key to IGF-1’s function lies in its binding to the IGF-1 receptor (IGF-1R), a transmembrane receptor similar to the IR with tyrosine kinase activity. This interaction is crucial for activating the PI3K/AKT pathway [81]. Notably, elevated IGF-1 levels have been associated with insulin resistance, and this complex interplay may contribute to the pathogenesis of DM2 [80].

In the reviewed articles, 16 studies exclusively employed molecular docking as the primary method for their in silico investigations [39,40,41,42,43,44,45,47,50,51,52,53,54,56,57,58]. The main functions of molecular docking are to determine the binding mode of a ligand to the protein, predict the binding affinity between the protein and ligand during compound optimization, and perform virtual screenings to identify potential drugs within large ligand databases [82].

This technique can be implemented using various docking methods. In rigid docking, both the ligand and receptor are treated as inflexible bodies. Alternatively, flexible docking considers the flexibility of both the ligand and receptor, allowing them to adjust their conformations to form an energetically optimal complex. Local move Monte Carlo sampling is another method that generates loop conformations by making small adjustments to the torsion angles of side chains and local movements of the loop [12]. Water docking explicitly includes structural water molecules in docking experiments, facilitating the formation of highly favorable hydrogen bonding networks between the ligand and the target binding site [83]. Although molecular docking is a widely used tool, it has some limitations, such as the restriction on the range of ligand and receptor conformations during pose prediction. Furthermore, the method may not respond adequately to the presence of ions or solvents, or pH or electric field. The scoring functions used may not correspond to experimentally observed binding affinities, and some interactions may be predicted with lower accuracy [84].

In contrast, four studies integrated both molecular docking and molecular dynamics to enhance the depth of their analysis [46,48,49,55]. These approaches allowed for a more comprehensive exploration of molecular interactions and dynamics within biological systems. The key findings from these in silico studies were systematically summarized, providing a clear overview of the potential applications and implications of these computational techniques in the field. This synthesis not only highlights the methodologies used but also underscores the significance of combining different in silico tools to achieve more robust and reliable results in molecular research [39,40,41,42,43,44,45,46,47,48,49,50,51,52,53,54,55,56,57,58].

In the studies that used molecular dynamics as a complementary in silico analysis, all successfully obtained the necessary information to understand the interaction between the receptor and ligand. They confirmed previously predicted binding sites and assessed the stability of the interaction over time [46,48,49,55]. Despite their accuracy, molecular dynamics simulations have limitations such as imprecision of force fields and high computational cost, which can lead to inadequate sampling of conformational states, restricting the ability to analyze and reveal functional properties of the analyzed systems [85]. To address these limitations, improved sampling algorithms are needed. Techniques such as replica-exchange molecular dynamics (REMD), which runs multiple replicas of a system simultaneously at different temperatures; metadynamics, which explores the free energy more extensively using computational resources to study biological conformations; and simulated annealing, which gradually reduces the system temperature during the simulation, can enhance the analysis [85,86,87].

The primary advantage of the studies that used molecular dynamics as a complementary in silico analysis was the ability to dynamically determine the interactions between ligands and targets over time, thereby identifying potential mechanisms of action that were later confirmed through experimental procedures. This approach produced consistent and corroborative results. These studies showed that the modulation of the therapeutic targets resulted in a secondary outcome: the direct improvement of biochemical parameters related to DM.

Although there is no consensus on the best in silico tool, the combination of molecular docking and molecular dynamics is widely used. Molecular dynamics stands out for its ability to more accurately represent the behavior of atoms within structures by simulating interactions over time, leading to more targeted and detailed predictions compared to molecular docking.

Upon analyzing the results of the included studies, it is evident that in silico investigations have made a significant contribution to exploring therapeutic targets for DM. Computational techniques serve as valuable tools for initial assessments and predictions, expanding the range of potential targets for future research. However, it is essential to recognize that in silico analyses should be complemented by in vivo and clinical studies to validate the predicted outcomes and ensure the relevance of these targets in the context of DM management.

Understanding the characteristics of the target and then searching for therapeutic agents is a widely employed practice, with structure-based drug design (SBDD) being one of the primary approaches in CADD [20,21]. The search for scientific evidence regarding promising targets for controlling DM facilitates a critical analysis of the acquired data and an assessment of the quality of the results. In addition, by verifying the reassessment of in vivo targets, it is possible to ensure the effectiveness of the targets to guide future studies.

The current study serves as a valuable resource for researchers investigating potential therapeutic options for DM. Its easy accessibility ensures efficient access to pertinent information. Furthermore, the therapeutic targets, identified through prior in silico investigations and subsequent in vivo validation, offer a strategic advantage. Researchers can now focus their efforts on targets with a higher likelihood of success, sparking innovation through the exploration of unconventional yet impactful avenues that align with their research goals.

Incorporating comprehensive insights into the mechanisms underlying these therapeutic targets establishes a robust foundation for developing new research strategies. This systematic document, carefully crafted through a rigorous methodology, serves as a guide for experimental endeavors. By offering valuable insights into the mechanisms associated with the therapeutic targets, it provides researchers with essential support for formulating effective research approaches.

These results are essential for the search for new therapeutic agents for the treatment of DM. They highlight potential therapeutic targets for this purpose, providing valuable insights for selecting targets to be investigated in in silico analyses. This information serves as a guide for future in vitro, in vivo, and clinical trials seeking alternatives in the treatment of DM.

According to the studies included in the SR, it became evident that numerous criteria associated with the risk of bias were categorized as uncertain. This uncertainty stemmed from the limited details provided in the in silico analyses, which, in turn, hindered reproducibility. The biases in describing methodological criteria may be linked to the relatively new nature of this approach. As observed in the included studies, the majority of publications date back only to the last five years.

According to the proposed risk of bias protocol, therapeutic targets should be selected based on their resolution, with a preference for those with a resolution of less than 2.5 Å. Resolutions above this threshold can complicate accurate atom positioning, potentially affecting the reliability of the results. Furthermore, it is recommended that the method used to obtain the structure of the therapeutic target be considered, with NMR spectroscopy being the most suitable as it generates a model that more closely resembles the biological one [31].

In addition to the care taken in selecting the therapeutic target, certain steps are proposed for ligand selection. These include filtering processes that optimize calculations and eliminate large molecules with low safety profiles or inadequate pharmacokinetic behavior. Another crucial step in optimizing ligand structure is the generation of all possible conformations characterized by minimum potential energy and geometric parameters, such as ideal bond lengths, angles, and dihedrals [31].

The choice of an appropriate tool for molecular docking is a significant concern in the bias domain. According to the study by Taldaev (2022) [31], the software considered the gold standard are GLIDE and GOLD. However, due to their cost, these tools may not be accessible to all studies, which could explain why most studies did not use them. In addition to being free-access software, Autodock (https://autodock.scripps.edu/) and its variations are also widely used in various molecular docking studies [88].

Therefore, it is crucial to utilize tools like the checklist developed by Taldaev (2022) [31] to comprehensively describe all methodological details involved in in silico studies. These criteria are essential for ensuring the quality and methodological rigor of such studies, and they should be provided to enable future bioinformatics analyses of higher quality.

As a result, this SR contributes to the advancement of clearer protocols in future studies and a more comprehensive description of articles employing this approach. The enhancement of data collection and analysis standardization will bring significant benefits to the entire field.

## 5. Conclusions

The primary objective of this SR was to identify therapeutic targets with significant potential for the treatment of DM through a combination of in silico analysis and subsequent in vivo validation. The development of this SR provides an overview that highlights a wide range of therapeutic targets suitable for DM treatment, as indicated by in silico studies corroborated through subsequent in vivo assessments, highlighting mainly the following targets: GLUT-4, DPP-IV, and PPARγ. The data also pointed out improvements in glycemic parameters.

The therapeutic targets identified in this review hold the potential for modulation through specific interactions with therapeutic agents. This modulation capability opens the door for the development of combination therapies designed to address multiple aspects of DM simultaneously. This strategy of combining therapies aims to enhance treatment effectiveness, offering a more comprehensive approach to disease management. Thus, the identified therapeutic targets not only provide individual intervention possibilities but also have the potential to play an integral role in a complex and multifaceted therapeutic approach.

In this sense, those targets can contribute to the discovery of new therapeutic agents for the treatment of DM, supporting future clinical studies with human beings where the following therapeutic targets have great potential to control glycemia in DM. In addition, another noteworthy aspect emphasized in this SR is the innovative potential to enhance methodological rigor, ultimately striving for higher quality and standardization of the information concerning the methodologies used in in silico studies. This effort aims to promote future research with a reduced risk of bias and, consequently, greater scientific evidence.

## Supplementary Materials

The following supporting information can be downloaded at: https://www.mdpi.com/article/10.3390/ijms25179213/s1.
