# Supplementary material for: In Silico Screening of Therapeutic Targets as a Tool to Optimize the Development of Drugs and Nutraceuticals in the Treatment of Diabetes mellitus: A Systematic Review
Source: Int J Mol Sci. 2024 Aug 25;25(17):9213. doi: 10.3390/ijms25179213 (PMC11394750; doi:10.3390/ijms25179213)
Supplement: Supplementary file 1 [file ijms-25-09213-s001.zip › ijms-3138053-supplementary.pdf]

**Table S1** Elements of the research question (What therapeutic targets have been used in *in silico* analyzes for the treatment of diabetes *mellitus*?) according to the PECo strategy (problem, exposure, and context) for systematic review.

| Description | Abbreviation | Question elements |
|---|---|---|
| Problem | P | Therapeutic targets used in the treatment of diabetes *mellitus* |
| Exposure | E | Diabetes *mellitus* |
| Context | Co | *In silico* studies with molecular dynamics and/or molecular docking |

**Table S2** Search equation for the databases used to include articles according to the eligibility criteria, aiming to answer the question: What therapeutic targets have been used in the *in silico* analyzes for the treatment of diabetes *mellitus*?

| Database | Search equation |
|---|---|
| Medline (PubMed) | ("therapeutic target" OR target OR treatment OR therapeutics) AND ("diabetes *mellitus*") AND ("*in silico*" OR "computer simulation") AND ("molecular dynamics simulation" OR "molecular dynamic" OR "molecular docking simulation" OR "molecular docking") |
| Web of Science | ("therapeutic target" OR target OR treatment OR therapeutics) AND ("diabetes *mellitus*") AND ("*in silico*" OR "computer simulation") AND ("molecular dynamics simulation" OR "molecular dynamic" OR "molecular docking simulation" OR "molecular docking") |
| Scopus<br>Filter used: Title/Abstract | (‘diabetes *mellitus*’) AND (‘molecular dynamics simulation’ OR ‘molecular dynamic’ OR ‘molecular docking simulation’ OR ‘molecular docking’) |
| Embase | ‘therapeutic target’/exp OR ‘therapeutic target’ OR target/exp OR target OR treatment/exp OR treatment OR therapy/exp OR therapy AND ‘diabetes *mellitus*’/exp OR ‘diabetes *mellitus*’ AND ‘*in silico* analysis’/exp OR ‘*in silico*’ OR ‘computer simulation’/exp OR ‘computer simulation’ OR ‘computer Model’/exp OR ‘computer Model’ AND ‘molecular dynamics’/exp OR ‘molecular dynamic’ OR ‘molecular docking’/exp OR ‘molecular docking’ |
| ScienceDirect<br>Filters used: Research articles and Title, Abstract, Key Words | (“diabetes *mellitus*”) AND (“*in silico*” OR “computer simulation” OR "molecular dynamics simulation" OR "molecular dynamic" OR "molecular docking simulation" OR "molecular docking") |
| Virtual Health Library<br>Filters used: Title, Abstract, Key Words | ("therapeutic target" OR target OR treatment OR therapeutics) AND ("diabetes *mellitus*") AND ("*in silico*" OR "computer simulation") AND ("molecular dynamics simulation" OR "molecular dynamic" OR "molecular docking simulation" OR "molecular docking") |

**Table S3** General characteristics of the structures of therapeutic targets *in silico* used in the included studies for the systematic review.

| Therapeutic target | Experimental Data Snapshot | | | Homology Modeling |
|---|---|---|---|---|
| | PDB ID | Method | Resolution | Protein Sequence |
| **DPP-IV** | 2ONC | X-Ray diffraction | 2.55 Å | NA |
| | 4A5S | X-Ray diffraction | 1.62 Å | NA |
| | 1R9N | X-Ray diffraction | 2.30 Å | NA |
| | 5Y7K | X-Ray diffraction | 2.51 Å | NA |
| **GLP-1R** | 3C5T | X-Ray diffraction | 2.10 Å | NA |
| **PPARγ** | 2PRG | X-Ray diffraction | 2.30 Å | NA |
| | 5TWO | X-Ray diffraction | 1.93 Å | NA |
| | 1I7I | X-Ray diffraction | 2.35 Å | NA |
| **Akt** | 3O96 | X-Ray diffraction | 2.70 Å | NA |
| | 3QKM | X-Ray diffraction | 2.2 Å | NA |
| | 2UZS | X-Ray diffraction | 2.46 Å | NA |
| **GLUT4** | NA | NA | NA | UNIPROT P14672 |
| | 4PYP | X-Ray diffraction | 3.17 Å | NA |
| | 5EQG | X-Ray diffraction | 2.90 Å | NA |
| | 3PCU | X-Ray diffraction | 2.00 Å | NA |
| **α-amilase** | 2QV4 | X-Ray diffraction | 1.97 Å | NA |
| **GLP-1** | 3IOL | X-Ray diffraction | 2.10 Å | NA |
| **GIP** | 2QKH | X-Ray diffraction | 1.90 Å | NA |
| **IR** | 1IRK | X-Ray diffraction | 2.10 Å | NA |
| **IRS1** | 1IRS | Solution nmr | NA | NA |
| **GSK3β** | 1Q5K | X-Ray diffraction | 1.94 Å | NA |
| **IGF1 and PI3K** (no information on the structure used) | --- | --- | --- | --- |

NA: Not applicable, PDB ID: Identification of the structure in the PDB database.
Glucose transporter 4 (GLUT-4), dipeptidyl peptidase IV (DPP-IV), peroxisome proliferator-activated receptor gamma (PPARγ), protein kinase B/AKT (AKT), glucagon-like peptide-1 (GLP-1), α-amylase, glucose-dependent insulinotropic polypeptide (GIP), insulin receptor substrate 1 (IRS1), glycogen synthase kinase 3 (GSK-3), insulin receptor (IR), phosphatidylinositol 3-kinase signaling pathway (PI3K), and insulin-like growth factor (IGF-1).

**Table S4** Characteristics of the *in silico* analyzes of the studies included in the systematic review.

| Therapeutic target | Therapeutic agent | *In silico* technique | Docking score or Binding energy | EPI or RMSD | Most interacting amino acids | Reference |
|---|---|---|---|---|---|---|
| **DPP-IV** (PDB 2ONC) | Naringin | Molecular docking | Pose 1: -56,89 (considering the complete structure of DPP-IV)<br><br>Pose 1: -63,27 (Considering single chain (A chain) outside the DPP-IV homotetramer structure)<br><br>Pose 1: -77,95; | --- | No information. | Parmar et al. (2012) [39] |

| | | | | | | |
|---|---|---|---|---|---|---|
| | | | (considering DPP-IV binding pocket(s)) | | | |
| **GLP-1R** (PDB 3C5T) | L-Glutamine | Molecular docking | **GLP-1R:** -6,87 | --- | **GLP-1R:** PHE-80, TYR-101, TRP-120, ASP-122, SER-124 and GLU-125 | Badole et al. (2013) [40] |
| **PPARγ** (PDB 2PRG)<br><br>**Akt** (PDB 3O96)<br><br>**GLUT4** (UNIPROT P14672) | Embelin | Molecular docking | **PPARγ:** − 8,18<br><br>**Akt:** − 5,30<br><br>**GLUT4:** - 6,44 | --- | **PPARγ:** LEU-228, ARG-288 and GLU-295.<br><br>**Akt:** VAL-271, GLN-79, THR-81, THR-82, and ASP-292<br><br>**GLUT4:** GLN-177, ASN-176, GLN-299. | Gandhi et al. (2013) [41] |
| **DPP-IV** (PDB 2ONC) | Glimepiride Sulfonamide (GS) | Molecular docking | **DPP-IV:**<br>Pose 1: -8,79;<br>Pose 2: -8,41;<br>Pose 3: -8,09;<br>Pose 4: -7,96;<br>Pose 5: -6,60. | --- | **DPP-IV:** ARG-669, PHE-357, LYS-554, TRP-629, TYR-631, TYR-662, TYR-666, GLY-632, GLU-206 and ARG-125. | Parmar et al. (2014) [42] |
| **PPARγ** (PDB 2PRG)<br><br>**GLUT4** (UNIPROT P14672) | Ficusin | Molecular docking | **PPARγ:** −7,04<br><br>**GLUT4:** -6,64 | --- | **PPARγ:** SER-289, HIS-323<br><br>**GLUT4:** ASP-427 | Irudayaraj et al. (2016) [43] |
| **PPARγ** (PDB 2PRG) | Isolated compounds of Indian traditional medicinal plants | Molecular docking | **3,3- (dimethyl allyl) halfordinol:** −8,5<br><br>**Cucurbitacin B:** -8,2<br><br>**Eremanthin:** -7,87<br><br>**Nymphayol:** -9,19<br><br>**Aurapten:** -7,6 | --- | **3,3-(dimethyl allyl) halfordinol:** ARG-288, TYR-473<br><br>**Cucurbitacin B:** GLU-291, SER-342, THR-268<br><br>**Eremanthin:** SER-342<br><br>**Nymphayol:** ARG-288 | Stalin et al. (2016) [a] [45] |

| | | | | | | |
|---|---|---|---|---|---|---|
| | | | | | **Aurapten:** GLN-286, HIS-449 | |
| **PPARγ** (PDB 2PRG)<br><br>**Akt** (PDB 3O96)<br><br>**GLUT4** (UNIPROT P14672) | 6-bromoembelin and vilangin | Molecular docking | 6-bromoembelin<br><br>**PPARγ:** - 5,60<br>**Akt:** -5,48<br>**GLUT4:** - 6,74<br><br>Vilangin<br><br>**PPARγ:** - 5,98<br>**Akt:** - 5,78<br>**GLUT4:** - 6,87 | --- | 6-bromoembelin<br><br>**PPARγ:** SER-289, HIS-323<br>**Akt:** SER-205, LYS-268<br>**GLUT4:** GLY-302, VAL-306, SER-310<br>Vilangin<br><br>**PPARγ:** GLU-343, SER-342<br>**Akt:** ASN-199, TRP-80, LEU-78, SER-216, GLN-59<br>**GLUT4:** GLU-136 | Stalin et al. (2016) [b] [44] |
| **α-amilase** (PDB 2QV4) | Taxifolin (TFN) | Molecular docking and molecular dynamics | **α-amylase:** 6,89 | Equilibrium was reached quickly after some initial fluctuations, which indicates a stable conformation for a nested structure. | **α-amylase:** TRP-59, TYR-62, GLN-63, ARG-195, GLU-233, HIE-299 and ASP-300 | Rehman et al. (2018) [46] |
| **GLP-1** (PDB 3IOL)<br><br>**GIP** (PDB 2QKH) | Phytochemicals from PTY-2: tuberostan, puererone, tuberosin, puererin, daidzin, | Molecular docking | **GLP-1**<br>Tuberostan: 8,15<br>Puererone: 7,34<br>Tuberosin: 7,85<br>Puererin: 6,76<br>Daidzin: 7,60<br><br>**Receptor GIP**<br>Tuberostan: 7,91<br>Puererone: 8,31 | --- | **GLP-1:**<br>Tuberostan: TYR- 69, LEU-89, TRP-91, LEU-123, CYS-126, GLU-127, GLU-128, TYR-19, GLN-23, LYS-26, ILE-29, ALA-30 e VAL-33.<br><br>**(Provided only this one for presenting better interaction)**<br><br>**Receptor GIP:**<br>Puererone: LYS-16, ILE- | Srivastava et al. (2018) [47] |

| | | | | | | |
|---|---|---|---|---|---|---|
| | | | Tuberosin: 7,53<br>Puererin: 7,61<br>Daidzin: 6,93 | | 17, GLN-19, GLN-20, ASP-21, ASN-24, GLN-30, PRO-89, TRP-90, ASN-120 e GLU-122<br><br>**(Provided only this one for presenting better interaction)** | |
| **IR**<br>(PDB 1IRK)<br><br>**GLUT-4**<br>(UNIPROT P14672) | β-sitosterol | Molecular docking and molecular dynamics | **IR:** - 10.96<br><br>**GLUT-4: -** 12.25 | Balance range from the beginning to the end of the simulation | **IR:** GLN-107 and GLN-444<br><br>**GLUT-4:** VAL-67, GLN-444, TYR-405, ARG-188, TYR-439 and TRP-173 | Ponnulakshmi et al. (2019) [48] |
| **IRS1**<br>(PDB 1IRS)<br><br>**AKT**<br>(PDB 3QKM) | β-sitosterol (SIT) | Molecular docking and molecular dynamics | **IRS-1:** -7,85<br><br>**AKT**: -7,60 | Complexes with IRS1 and AKT did not show much fluctuation over the 50ns period. | **IRS1:** LYS-161, SER-189 and ALA-259.<br><br>**AKT:** THR-211 and GLU-228 | Babu et al. (2020) [49] |
| **PPARγ**<br>(PDB 5TWO)<br><br>**DPP-IV**<br>(PDB 4A5S) | Thymoquinone (2-isopropyl-5-methylbenzo-1,4 quinone) (TQ) | Molecular docking | **PPARγ:** -5,7<br><br>**DPP-IV:** -6,3 | --- | **PPARγ:** ILE-262, LYS-263, GLY-284, CYS-285, ARG-288, LEU-330, LEU-333, LEU-340, ILE-341 and SER-342<br><br>**DPP-IV:** TYR-547, SER-630, TYR-631, VAL-656, TRP-659, TYR-662, TYR-666 and VAL-711 | Alshahrani (2021) [50] |
| **GLUT 4**<br>(PDB 4PYP) | Stevioside | Molecular docking | **GLUT 4:** -7,8 | --- | **GLUT 4:** GLN-113, TYR-168, TRP-173, ARG-188, ALA-190, ARG-336, GLU-359 GLY-404, | Deenadayalan et al. (2021) [51] |

| | | | | | | |
|---|---|---|---|---|---|---|
| | | | | | TYR-405 and ARG-433 | |
| **GSK3β** (PDB 1Q5K)<br><br>**PPARγ** (PDB 1I7I)<br><br>**DPPIV** (PDB 1R9N)<br><br>**GLUT 4** (PDB 5EQG) | Pterostilbene and Androgr apholide | Molecular docking | Pterostilbene<br><br>**GSK3β:** -7,51<br><br>**PPARγ:** -7,71<br><br>**DPPIV:** -7,0<br><br>**GLUT 4:** -7,13<br><br>Andrographolid e<br><br>**GSK3β:** - 7,17<br><br>**PPARγ:** - 9,33<br>**PPARα:** - 5,84<br><br>**DPPIV:** - 6,64<br><br>**GLUT 4:** - 9,36 | --- | Pterostilbene<br><br>**GSK3β:** VAL-135, LYS-85<br><br>**PPARγ:** TYR-473, HIS-449, ARG-288<br><br>**DPPIV:** SER-630, HIS-740, GLU-206<br><br>**GLUT 4:** GLU-396, ASN-431<br>Andrographoli de<br><br>**GSK3β:** VAL-135 and VAL-61<br><br>**PPARγ:** TYR-327, ARG-288 and ARG-288<br><br>**DPPIV:** PHE-357, ARG-358, GLU-205, ARG-358 and ARG-358<br><br>**GLUT 4:** GLU-396, ASN-304, GLN-298, ASN-333, GLN-298 and TRP-404 | Dound et al. (2021) [52] |
| **DPP-4** (PDB 5Y7K) | Phytoconstit uents of W. coagulans fruit:<br><br>Withanolide D<br><br>Sitoindoside IX<br><br>Withanone<br><br>Withanolide B | Molecular docking | **Withanolide D:** – 9.2<br><br>**Sitoindoside IX:** -9,8<br><br>**Withanone:** -7,9<br><br>**Withanolide B:** -9,5<br><br>**Withaferine A:** -8,1 | --- | **Withanolide D:** VAL-207<br><br>**Sitoindoside IX:** GLU-205, HIS-740 and TYR-547<br><br>**Withanone:** ARG-125, TYR-662 and VAL-656 | Ram et al. (2021) [53] |

| | | | | | | |
|---|---|---|---|---|---|---|
| | Withaferine A<br><br>Withasomnine<br><br>Withangulatin A<br><br>Withacoagulin H<br><br>Withanolide E | | **Withasomnine:** -6,6<br><br>**Withangulatin A:** -8,8<br><br>**Withacoagulin H:** -8,9<br><br>**Withanolide E:** -7,6 | | **Withanolide B:** TYR-547 and HIS-740<br><br>**Withaferine A:** SER-209<br><br>**Withasomnine:** GLU-206<br><br>**Withangulatin A:** SER-209, ARG-125, GLU-205, GLU-206 and TYR-662<br><br>**Withacoagulin H:** GLU-206, SER-209, TYR-547, GLU-205 and ASP-663<br><br>**Withanolide E:** GLU-206, SER-209, ASN-710 and HIS-740 | |
| **IGF1, PI3K and AKT** (no information on the structure used) | Pentanoic acid, butyric acid and propionic acid | Molecular docking | **Pentanoic acid with IGF1:** around 4.25<br><br>**Butyric acid with PI3K:** around 4.25<br><br>**Propionic acid with AKT:** around 4.25 | --- | **Pentanoic acid with IGF1:** GLY-30 and SER-33<br><br>**Butyric acid with PI3K:** GLN-846, ARG-849 and CYS-869<br><br>**Propionic acid with AKT:** LYS-179 and GLU-198 | Xia et al. (2021) [54] |
| **DPP-4** (PDB 5Y7K) | Withania coagulans fruit phytochemicals:<br><br>Withacoagin<br><br>Withasomnine<br><br>Withanolide E | Molecular docking and molecular dynamics | **Withacoagin:** -9,2<br><br>**Withasomnine:** -6,6<br><br>**Withanolide E:** -7,6 | Complexes undergo minimal changes during simulations, but the complex with Withasomnine balanced out fastest among all three. | **Withacoagin:** TYR-547<br><br>**Withasomnine:** GLU-206<br><br>**Withanolide E:** GLU-206, SER-209, ASN-710 and HIS-740 | Kumar et al. (2022) [55] |

| | | | | | | |
|---|---|---|---|---|---|---|
| **GLUT 4** (PDB 3PCU) | Phytoconstituents of the butanol fraction of B. ferrugínea: pyrogallol and sitosterol | Molecular docking | **Pyrogallol:** -5,0<br><br>**Sitosterol:** −7,9 | --- | **Pyrogallol:** ILE-21, ALA-24, ALA-25, GLN-28, LEU-62, PHE-66, ARG-69, LEU-79, ALA-80 and THR-81<br><br>**Sitosterol:** VAL-18, ILE-21, CYS-22, ALA-24, ALA-25, GLN-28, TRP-58, LEU-62, ILE-63, PHE-66, ARG-69, ILE-77, LEU-78, LEU-79, ALA-80, VAL-95, ILE-98, PHE-99, CYS-185, HIS-188, LEU-189 and PHE-192 | Oyebode et al. (2022) [56] |
| **AKT1** (PDB 2UZS) | Berberine (BBR)+ Paeoniflorin (PF) | Molecular docking | **BBR:** -7,2<br><br>**PF:** -6,1 | --- | **BBR:** GLU-91<br><br>**PF:** LYS-14, LYS-17, ARG-25, ASN-54 and ARG-86 | Zhang et al. (2022) [57] |
| **AKT** (no information on the structure used) | Beta-Sitosterol as a component of JinXiaoXiao Ke (JXXKD) | Docking molecular | -7,3 | --- | GLU-91 | Lin et al. (2023) [58] |

Glucose transporter 4 (GLUT-4), dipeptidyl peptidase IV (DPP-IV), peroxisome proliferator-activated receptor gamma (PPARγ), protein kinase B/AKT (AKT), glucagon-like peptide-1 (GLP-1), α-amylase, glucose-dependent insulinotropic polypeptide (GIP), insulin receptor substrate 1 (IRS1), glycogen synthase kinase 3 (GSK-3), insulin receptor (IR), phosphatidylinositol 3-kinase signaling pathway (PI3K), and insulin-like growth factor (IGF-1).

[a] Study 1 by Stalin et al. (2016)

[b] Study 2 by Stalin et al. (2016)

**Table S5** Characteristics of in vivo reassessments of included studies included in the systematic review.

| Therapeutic target | In vivo model | Therapeutic agent administered in vivo | In vivo therapeutic target analysis | Results of analysis of in vivo therapeutic targets | Potential Impact for Applicability in Diabetes Treatment | Reference |
|---|---|---|---|---|---|---|

| | | | | | | |
|---|---|---|---|---|---|---|
| **DPP-IV** | Healthy male albino Wistar rats | Naringin | DPP-IV assay in serum samples | Significantly lower level of serum DPP-IV activity [1]<br><br>(Compared to a control group of untreated healthy rats [1]) | Lower glucose level and higher insulin level [1]<br><br>(Compared to a control group of untreated healthy rats [1]) | Parmar et al. (2012) [39] |
| **GLP-1R** | Male Sprague Dawley Rats with Streptozotocin-Nicothiamide-Induced DM (STZ-NTM) | L-Glutamine | Analysis of GLP-1 active amide concentration in plasma and colon, and during the oral glucose tolerance test<br><br>Analysis of GLP-1 mRNA expression from intestinal tissue | Significantly higher level of GLP-1 active amide in plasma and colon and during the oral glucose tolerance test [2]<br><br>Increased GLP-1 mRNA expression [2]<br><br>(Compared to control group with untreated diabetes [2]) | Significantly lower level of glucose, glycated hemoglobin and higher plasma and pancreatic insulin [2]<br><br>(Compared to control group with untreated diabetes [2]) | Badole et al. (2013) [40] |
| **PPARγ**<br><br>**Akt**<br><br>**GLUT4** | Male Wistar rats with streptozotocin-induced DM (STZ) and fed High Fat Diet, cholesterol solution in coconut oil | Embelin | mRNA expression analysis and total proteins of PPAR γ, Akt, and GLUT4 from adipose tissue, liver, and skeletal muscle | Increased expression of PPAR γ mRNA and total proteins in adipose tissue, liver and skeletal muscle [2]<br><br>Increased Akt and GLUT4 mRNA gene expression in adipose tissue [2]<br><br>(Compared to control group with untreated diabetes [2]) | Improvement in glucose tolerance levels, significant reduction in serum glucose, improvement in plasma insulin level [2]<br><br>(Compared to control group with untreated diabetes [2]) | Gandhi et al. (2013) [41] |
| **DPP-IV** | Wistar albino rats with streptozotocin-induced DM (STZ) | Glimepiride Sulfonamide (GS) | DPP-IV assay in serum samples | Significantly lower level of serum DPP-IV activity [1]<br><br>(Compared to the control group of untreated healthy rats [1]) | Significantly lower level of glucose and insulin [1]<br><br>(Compared to the control group of untreated healthy rats [1]) | Parmar et al. (2014) [42] |

| | | | | | | |
|---|---|---|---|---|---|---|
| **PPARγ** **GLUT4** | Male Wistar rats with streptozotocin-induced DM (STZ) fed a high-fat diet | Ficusin | Analysis of PPARγ and GLUT4 mRNA expression in adipose tissue | Increased PPARγ and GLUT4 mRNA expression [2] (Compared to control group with untreated diabetes [2]) | Significantly lower glucose level, better oral glucose tolerance, significantly higher insulin level and glycogen content, considering high doses of the therapeutic agent [2] (Compared to control group with untreated diabetes [2]) | Irudayaraj et al. (2016) [43] |
| **PPARγ** | Male Wistar albino rats with streptozotocin-induced DM (STZ) | Nymphayol | Analysis of PPARγ mRNA expression from adipose tissue, liver, and skeletal muscle | Significantly higher PPARγ mRNA expression in adipose tissue and slightly increased expression in skeletal muscle. There was no difference in liver [2] (Compared to control group with untreated diabetes [2]) | Improved oral glucose tolerance [2] (Compared to control group with untreated diabetes [2]) | Stalin et al. (2016) [a] [45] |

| | | | | | | |
|---|---|---|---|---|---|---|
| **PPARγ**<br><br>**Akt**<br><br>**GLUT4** | Male Wistar albino rats with streptozotocin-induced DM (STZ) | 6-bromoembelin and vilangin | Analysis of PPARγ, Akt and GLUT4 mRNA and total protein expression in adipose tissue, liver and skeletal muscle | Increased expression of mRNA and total proteins of PPARγ, Akt and GLUT4 in liver and skeletal muscle [2]<br><br>(Compared to control group with untreated diabetes [2]) | Statistically lower glucose level and improved glucose tolerance [2]<br><br>Lower plasma insulin level [1]<br><br>(Compared to the control group of untreated healthy rats [1])<br><br>(Compared to control group with untreated diabetes [2]) | Stalin et al. (2016) [b] [44] |
| **α-amilase** | Albino rats of both sexes with alloxan-induced DM | Taxifolin (TFN) | α-amylase serum level analysis | Statistically lower level of α-amylase when administering high doses of the therapeutic agent [2]<br><br>(Compared to control group with untreated diabetes [2]) | Statistically lower blood glucose level [2]<br><br>(Compared to control group with untreated diabetes [2]) | Rehman et al. (2019) [46] |
| **GLP-1**<br><br>**GIP** | Male Charles Foster rats with streptozotocin-induced DM (STZ) | PTY-2 | Plasma analysis of GLP-1 and GIP<br><br>Analysis of GLP-1R and GIP-R mRNA expression in pancreatic tissue | Statistically higher levels of GLP-1 and GIP [2]<br><br>Increased GLP-1R and GIP-R mRNA expression [2]<br><br>(Compared to control group with untreated diabetes [2]) | Statistically lower levels of glucose. Greater perimeter and number of islet cells in Langerhans. Significantly higher level of Insulin and Bcl 2 protein expression in the islets of Langerhans [2]<br><br>(Compared to control group with untreated diabetes [2]) | Srivastava et al. (2018) [47] |
| **IR**<br><br>**GLUT-4** | Male Wistar albino rats | β-sitosterol | Analysis of the expression of adipose tissue IR | Higher levels of IR protein and GLUT-4 [2] | Improved glucose and insulin tolerance, lower | Ponnulakshmi et al. (2019) [48] |

| | | | | | | |
|---|---|---|---|---|---|---|
| | with DM induced by a high-fat diet and 30% sucrose in drinking water | | proteins and GLUT-4 in the cytosol | (Compared to control group with untreated diabetes [2]) | fasting glucose levels [2]<br><br>(Compared to control group with untreated diabetes [2]) | |
| **IRS1**<br><br>**AKT** | Male Wistar albino rats with DM induced by a high-fat diet and 30% sucrose in drinking water | β-sitosterol (SIT) | Analysis of mRNA expression and total proteins of IRS-1 and AKT from adipose tissue | Significantly higher expression of mRNA and total proteins of IRS-1 and AKT [2]<br><br>(Compared to control group with untreated diabetes [2]) | Significant higher expression of mRNA and total proteins of GLUT4, as well as improvement of serine/thionine phosphorylation of Akt [2].<br><br>Improved IR mRNA expression [2,3]<br><br>(Compared to control group with untreated diabetes [2])<br><br>(Compared to DM group treated with standard drug [3]) | Babu et al. (2020) [49] |
| **PPARγ**<br><br>**DPP-IV** | Male Wistar rats with streptozotocin-induced DM (STZ) fed a high-fat diet | Thymoquinone (2-isopropyl-5-methylbenzo-1,4 quinone) (TQ) | Nuclear protein analysis (adipose tissue) to measure PPARγ and DPP-IV serum analysis | Higher expression of PPARγ in adipose tissue and lower of DPP-IV [2]<br><br>(Compared to control group with untreated diabetes [2]) | Significantly lower level of glucose, insulin and HOMA-IR, improvement in OGTT [2]<br><br>(Compared to control group with untreated diabetes [2]) | Alshahrani (2021) [50] |
| **GLUT 4** | Male Wistar rats with DM induced by a high-fat diet and 30% sucrose in drinking water | Stevioside | Muscle GLUT 4 mRNA expression analysis | Increased GLUT 4 mRNA expression [2]<br><br>(Compared to control group with untreated diabetes [2]) | Reduction of elevated fasting glucose levels and improved glucose tolerance [3]<br><br>Restoration of insulin levels [1]<br><br>Increased IR gene expression in muscles [2]<br><br>(Compared to a control group of untreated healthy rats [1]) | Deenadayalan et al. (2021) [51] |

| | | | | | | |
|---|---|---|---|---|---|---|
| | | | | | (Compared to control group with untreated diabetes [2])<br><br>(Compared to DM group treated with standard drug [3]). | |
| **GSK3β**<br><br>**PPARγ**<br><br>**DPPIV**<br><br>**GLUT 4** | Male Wistar albino rats with streptozotocin-induced DM (STZ) | A blend of Ptyrone™, containing combination of hydroalcoholic extract of Andrographis paniculata Nees. and Pterocarpus marsupium Roxb. | Analysis of GSK3β, PPARγ and GLUT 4 mRNA expression in liver tissue and DPP-IV assay in plasma samples | Increased expression of PPARγ, GSK3β and GLUT4. No significant inhibition of DPP4 [2]<br><br>(Compared to control group with untreated diabetes [2]) | Lower fasting blood glucose level [2].<br><br>(Compared to control group with untreated diabetes [2]) | Dound et al. (2021) [52] |
| **DPP-4** | Wistar rats with dexamethasone-induced DM fed a sucrose-rich diet along with carbohydrate-rich foods | Ethanol extracts of W. coagulans fruit | DPP-IV assay in serum samples | Statistically low levels of DPP-4 [1,2]<br><br>(Compared to a control group of untreated healthy rats [1])<br><br>(Compared to control group with untreated diabetes [2]) | Statistically low levels of glucose, insulin, insulin resistance. Statistically increased levels of β-cell function and insulin sensitivity [1,2]<br><br>Statistically increased levels in islet cell mass in pancreatic tissues and restoration of vascular tissues [2]<br><br>(Compared to a control group of untreated healthy rats [1])<br><br>(Compared to control group with untreated diabetes [2]) | Ram et al. (2021) [53] |
| **IGF1, PI3K and AKT** | Male C57BL/6J mice with DM induced by a high-fat diet followed by | CS polysaccharides (CSP) | Analysis of IGF1, PI3K, and AKT mRNA expression.<br><br>Immunohistochemical analysis of IGF1 and AKT expression in the colon | Increased expression of IGF1, PI3K, and AKT mRNA [2]<br><br>Significantly higher expression of IGF1 and | Lower fasting glucose levels, and rapid decrease in glucose spikes during the OGTT test [2].<br><br>Serum insulin levels close to | Xia et al. (2021) [54] |

| | | | | | | |
|---|---|---|---|---|---|---|
| | streptozotocin (STZ) | | | AKT in the colon [2]<br><br>(Compared to control group with untreated diabetes [2]) | those of untreated healthy control animals [1].<br><br>(Compared to a control group of untreated healthy rats [1])<br><br>(Compared to control group with untreated diabetes [2]) | |
| **DPP-4** | Male Wistar albino rats with streptozotocin-induced DM (STZ) | Extract of Withania coagulans | DPP-IV assay in serum samples | Statistically low levels of DPP-4 [1,2]<br><br>(Compared to a control group of untreated healthy rats [1])<br><br>(Compared to control group with untreated diabetes [2]) | Statistically low level of glucose, insulin and HOMA indexes [1,2]<br><br>Regeneration and restoration of the islets of Langerhans [2]<br><br>(Compared to a control group of untreated healthy rats [1])<br><br>(Compared to control group with untreated diabetes [2]) | Kumar et al. (2022) [55] |
| **GLUT 4** | Male Sprague Dawley rats with DM induced by fructose (10%) followed by streptozotocin (STZ) | Bridelia ferruginea extract | Analysis of GLUT 4 mRNA molecular expression in liver and muscle | Increased GLUT 4 mRNA expression [2]<br><br>(Compared to control group with untreated diabetes [2]) | Higher levels of liver glycogen when administering high doses of the therapeutic agent [3]<br><br>(Compared to DM group treated with standard drug [3]) | Oyebode et al. (2022) [56] |
| **AKT1** | Male C57BL/6J mice with high-fat diet-induced DM | Berberine (BBR)+ Paeoniflorin (PF) | Western blot analysis with primary AKT1 monoclonal antibodies in liver tissues | Higher levels of AKT1 phosphorylation [2]<br><br>(Compared to control group with untreated diabetes [2]) | Lower fasting glucose levels, lower OGTT glucose spikes [2]<br><br>(Compared to control group with untreated diabetes [2]) | Zhang et al. (2022) [57] |
| **AKT** | Male Goto-Kakizaki (GK) rats with DM induced by a high- | JinXiaoXiaoKe Decoction (JXXKD) | AKT mRNA analysis | Statistically low levels of AKT expression [4] | Lower Fasting Glucose Levels and HOMA-IR [4]<br><br>(Compared to the untreated model group [4]) | Lin et al. (2023) [58] |

| | | | | | | |
|---|---|---|---|---|---|---|
| | sugar, high-fat diet | | | (Compared to the untreated model group [4]) | | |

[1] These data are related to the test group compared to the untreated healthy control group.
[2] These data are related to the test group compared to the control group with untreated diabetes.
[3] These data are related to the test group compared to the control group with diabetes treated with the standard drug.
[4] These data are related to the test group compared to the group fed a high-sugar and high-fat diet during the experiment.

Glucose transporter 4 (GLUT-4), dipeptidyl peptidase IV (DPP-IV), peroxisome proliferator-activated receptor gamma (PPARγ), protein kinase B/AKT (AKT), glucagon-like peptide-1 (GLP-1), α-amylase, glucose-dependent insulinotropic polypeptide (GIP), insulin receptor substrate 1 (IRS1), glycogen synthase kinase 3 (GSK-3), insulin receptor (IR), phosphatidylinositol 3-kinase signaling pathway (PI3K), and insulin-like growth factor (IGF-1).

[a] Study 1 by Stalin et al. (2016).
[b] Study 2 by Stalin et al. (2016).
